# Supplementary material for: Mapping the determinants of catalysis and substrate specificity of the antibiotic resistance enzyme CTX-M β-lactamase
Source: Commun Biol. 2023 Jan 12;6:35. doi: 10.1038/s42003-023-04422-z (PMC9837174; doi:10.1038/s42003-023-04422-z)
Supplement: Supplementary file 2 — Supplementary Information [file 42003_2023_4422_MOESM2_ESM.pdf]

Supporting Information

Figure S1. X-ray crystal structure of CTX-M-14 β-lactamase with key active site structural motifs displayed.

Figure S2. Fitness values conferred to *E. coli* in the presence of ampicillin by mutant CTX-M-14 enzymes.

Figure S3. Fitness values conferred to *E. coli* in the presence of cefotaxime by mutant CTX-M-14 enzymes.

Figure S4. Fitness values conferred to *E. coli* in the presence of ceftazidime by mutant CTX-M-14 enzymes.

Figure S5. Michaelis-Menten plots used to determine steady state kinetic parameters.

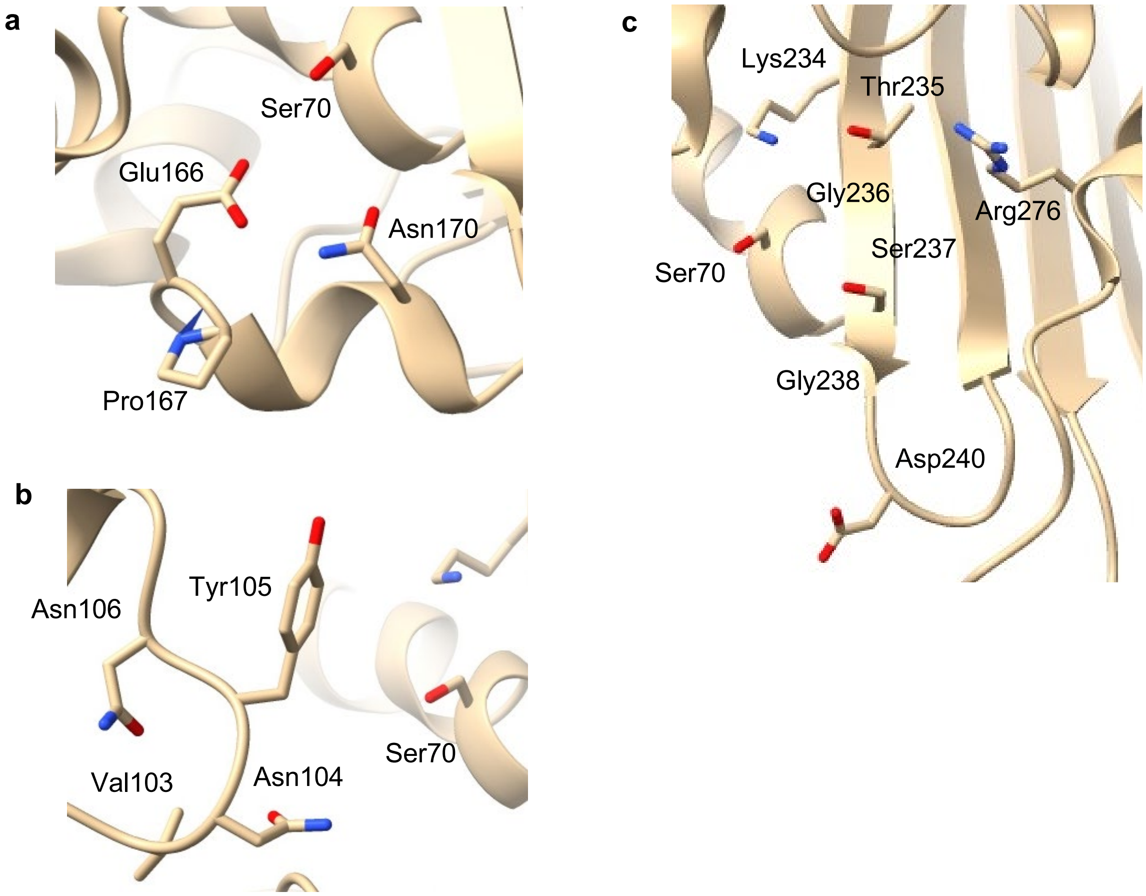

Figure S1. Key structural motifs of CTX-M-14 β-lactamase (PDB: 1YLT), including **(a)** the omega loop containing residues 166-170, **(b)** the 103-106 loop, and **(c)** the β3 strand containing residues 234-240.

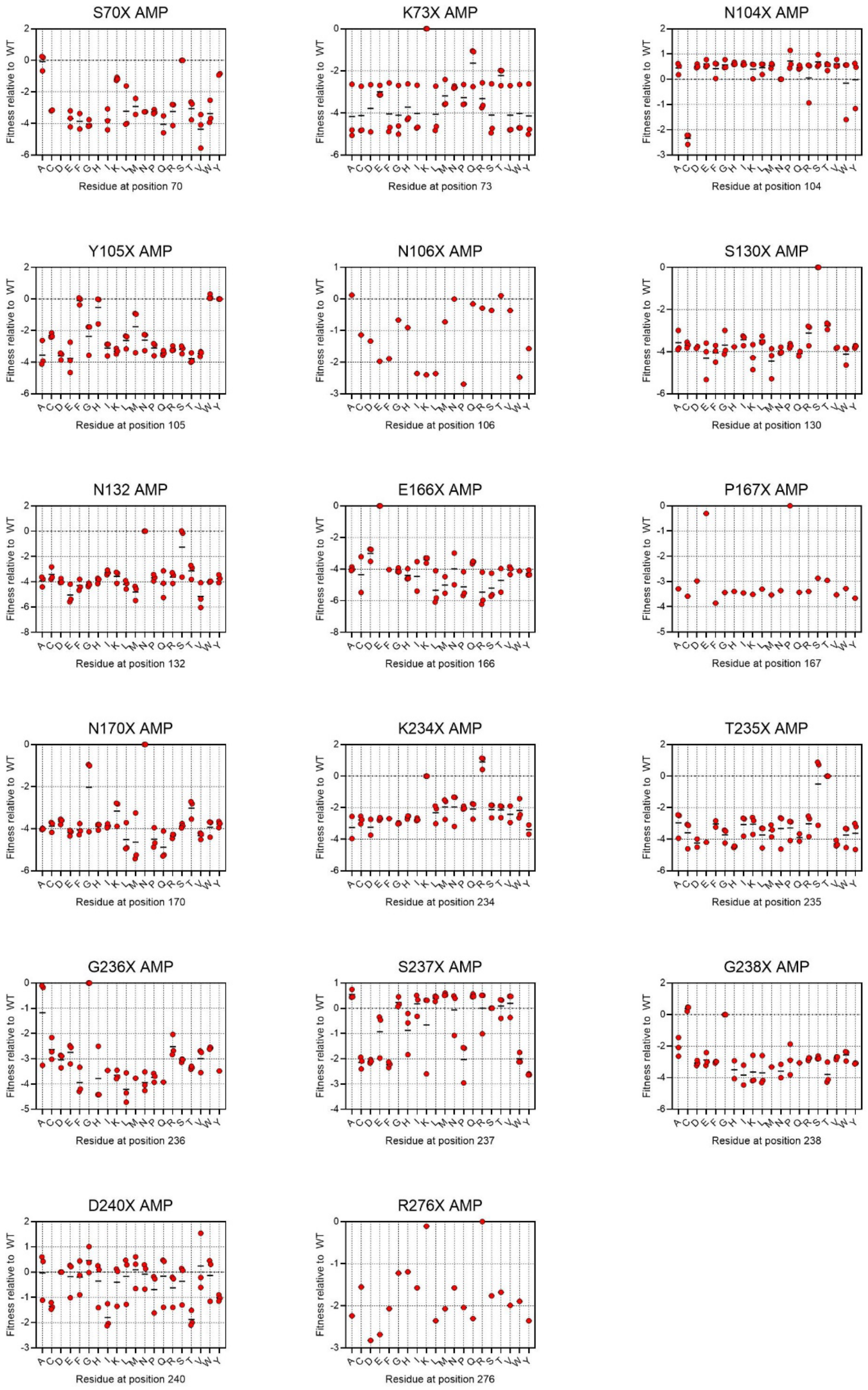

Figure S2. Individual biological replicates are shown, which represent the fitness conferred by each mutant to *E. coli* for growth in the presence of ampicillin (AMP) for each residue position. In cases where the frequency of the mutant was equal to 0 following selection, the replicate was excluded. In cases where all replicates were excluded, the fitness was not quantifiable and therefore not included on the graph, although we can conclude the fitness is very low.

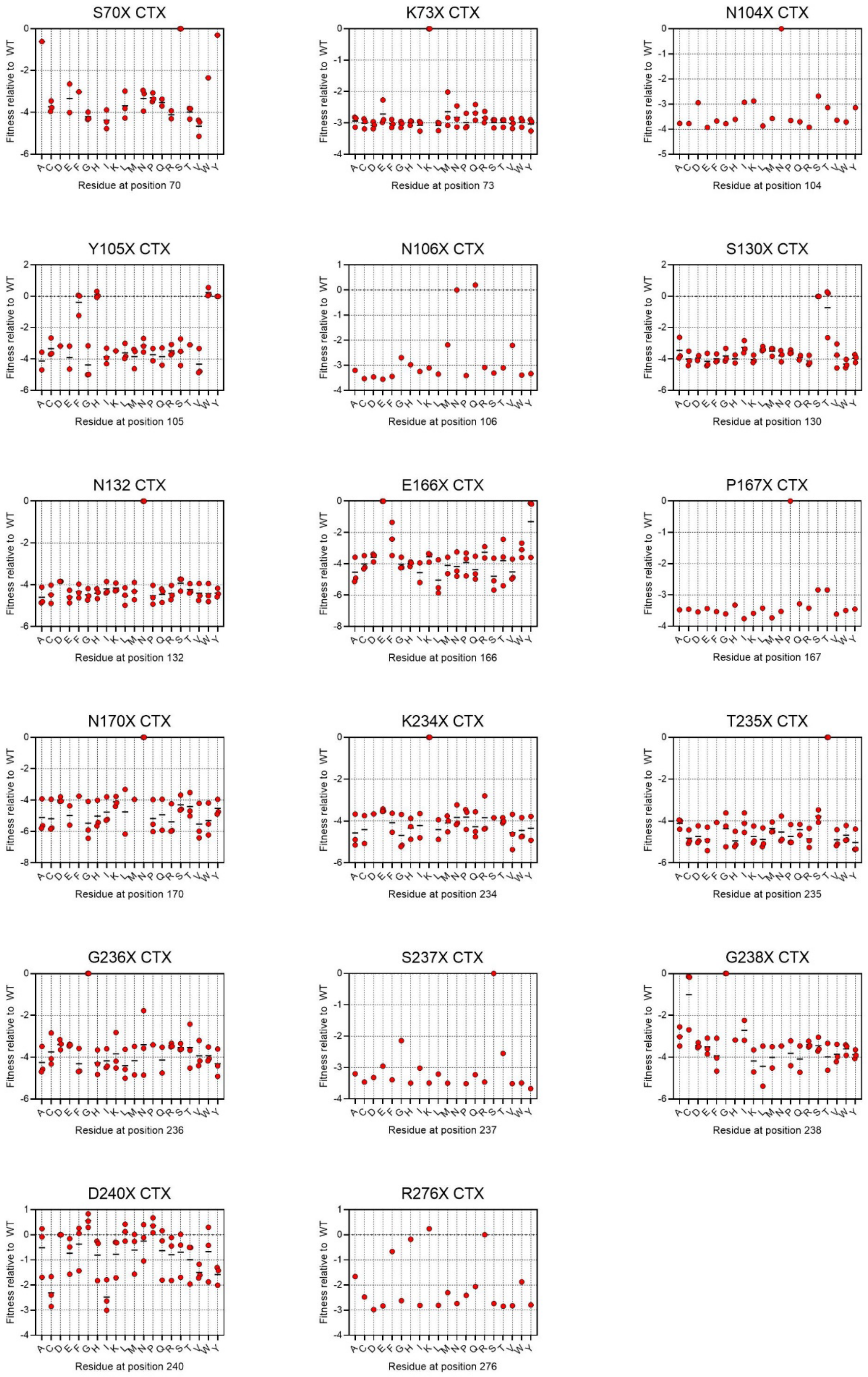

Figure S3. Individual biological replicates are shown, which represent the fitness conferred by each mutant to *E. coli* for growth in the presence of cefotaxime (CTX) for each residue position. In cases where the frequency of the mutant was equal to 0 following selection, the replicate was excluded. In cases where all replicates were excluded, the fitness was not quantifiable and therefore not included on the graph, though we can conclude the fitness is very low.

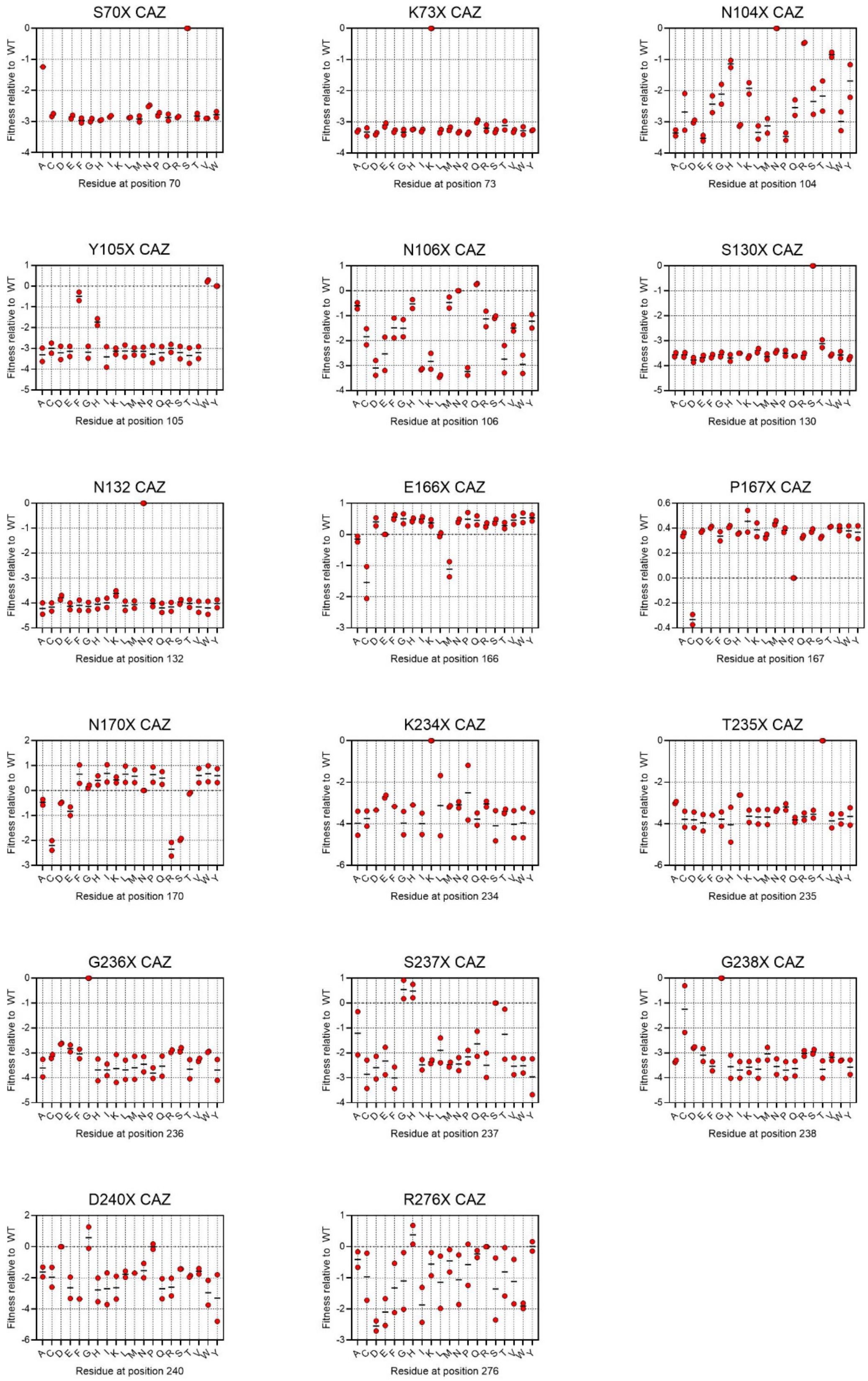

Figure S4. Individual biological replicates are shown, which represent the fitness conferred by each mutant to *E. coli* for growth in the presence of ceftazidime (CAZ) for each residue position. In cases where the frequency of the mutant was equal to 0 following selection, the replicate was excluded. In cases where all replicates were excluded, the fitness was not quantifiable and therefore not included on the graph, though we can conclude the fitness is very low.

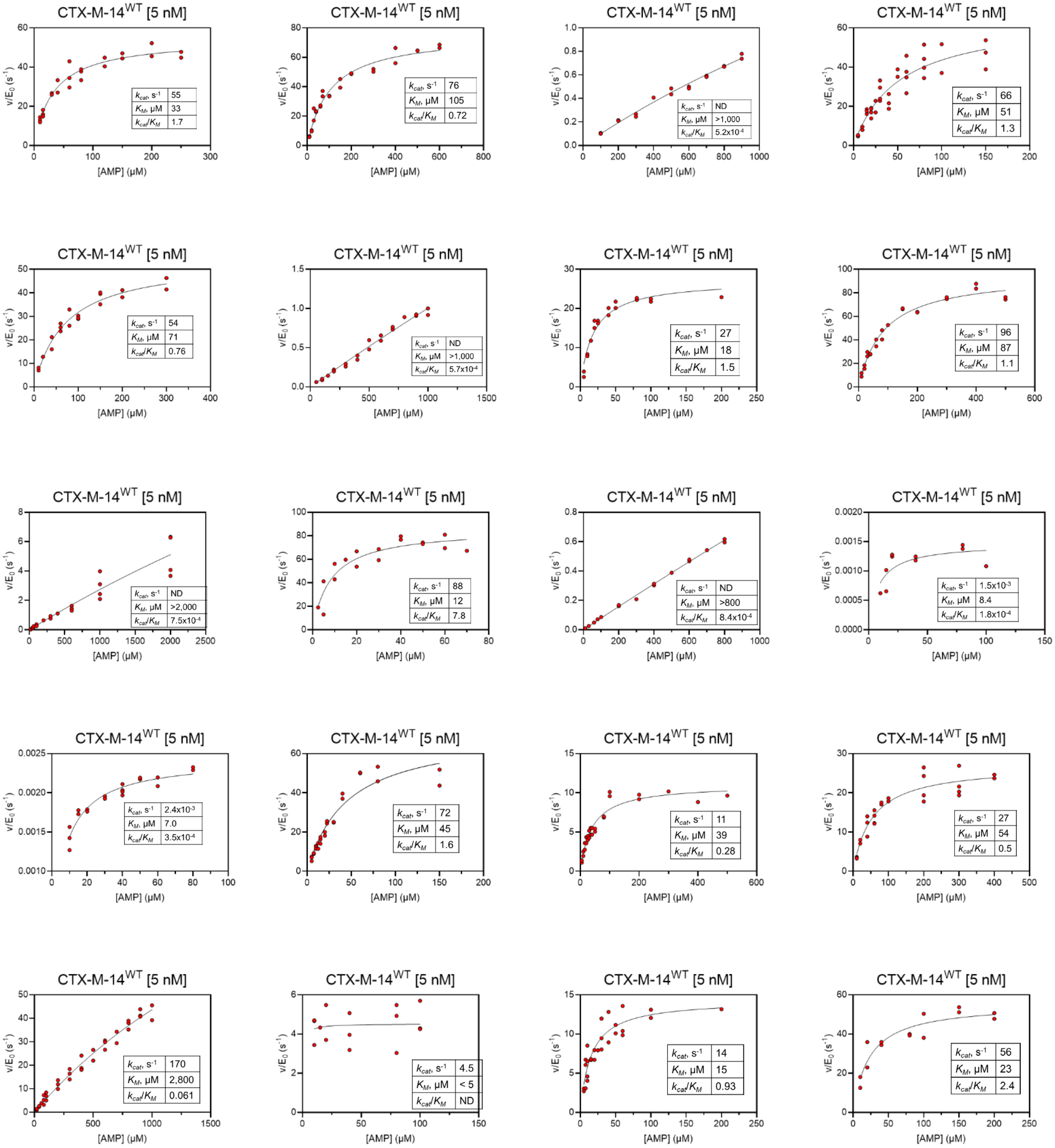

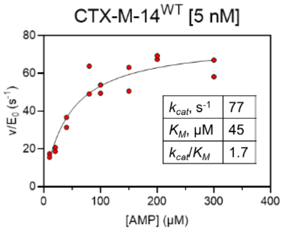

Figure S5. Each curve was fitted to the Michaelis-Menten model in Graphpad Prism to determine steady-state kinetic parameters (k_{cat}, K_M, and k_{cat}/K_M) described in Tables 2 and 3. Mutant CTX-M-14 enzymes were tested against cefotaxime (CTX), ampicillin (AMP), or ceftazidime (CAZ).
