# Supplementary material for: Mapping the determinants of catalysis and substrate specificity of the antibiotic resistance enzyme CTX-M β-lactamase
Source: Commun Biol. 2023 Jan 12;6:35. doi: 10.1038/s42003-023-04422-z (PMC9837174; doi:10.1038/s42003-023-04422-z)
Supplement: Supplementary file 3 — Description of Additional Supplementary Files [file 42003_2023_4422_MOESM3_ESM.pdf]

# Description of Additional Supplementary Files

File name: Supplementary Data 1

Description: Sequencing counts used to calculate CTX-M-14 variant fitness

File name: Supplementary Data 2

Description: The source data behind graphs in the paper
