# Supplementary material for: Mapping the determinants of catalysis and substrate specificity of the antibiotic resistance enzyme CTX-M β-lactamase
Source: Commun Biol. 2023 Jan 12;6:35. doi: 10.1038/s42003-023-04422-z (PMC9837174; doi:10.1038/s42003-023-04422-z)
Supplement: Supplementary file 6 — Reporting Summary [file 42003_2023_4422_MOESM6_ESM.pdf]

nature portfolio

Corresponding author(s): Dr. Timothy Palzkill

Last updated by author(s): Dec 15, 2022

Reporting Summary

Nature Portfolio wishes to improve the reproducibility of the work that we publish. This form provides structure for consistency and transparency in reporting. For further information on Nature Portfolio policies, see our Editorial Policies and the Editorial Policy Checklist.

Statistics

For all statistical analyses, confirm that the following items are present in the figure legend, table legend, main text, or Methods section.

n/a	Confirmed	
☐	☒	The exact sample size (*n*) for each experimental group/condition, given as a discrete number and unit of measurement
☐	☒	A statement on whether measurements were taken from distinct samples or whether the same sample was measured repeatedly
☒	☐	The statistical test(s) used AND whether they are one- or two-sided<br>*Only common tests should be described solely by name; describe more complex techniques in the Methods section.*
☒	☐	A description of all covariates tested
☐	☒	A description of any assumptions or corrections, such as tests of normality and adjustment for multiple comparisons
☐	☒	A full description of the statistical parameters including central tendency (e.g. means) or other basic estimates (e.g. regression coefficient) AND variation (e.g. standard deviation) or associated estimates of uncertainty (e.g. confidence intervals)
☒	☐	For null hypothesis testing, the test statistic (e.g. *F*, *t*, *r*) with confidence intervals, effect sizes, degrees of freedom and *P* value noted<br>*Give P values as exact values whenever suitable.*
☒	☐	For Bayesian analysis, information on the choice of priors and Markov chain Monte Carlo settings
☒	☐	For hierarchical and complex designs, identification of the appropriate level for tests and full reporting of outcomes
☒	☐	Estimates of effect sizes (e.g. Cohen's *d*, Pearson's *r*), indicating how they were calculated

Our web collection on statistics for biologists contains articles on many of the points above.

Software and code

Policy information about availability of computer code

Data collection	*Provide a description of all commercial, open source and custom code used to collect the data in this study, specifying the version used OR state that no software was used.*
Data analysis	Analysis of NGS deep sequencing FASTQ files was performed using a custom Python 3.0 script. DNA sequence reads were filtered for a minimum PHRED score of 20 for each nucleotide across the read using Trimmomatic (Bolger et. al. 2014). The selection condition of each read was determined based on the 8-bp barcode, and the codon at the randomized position was identified by the 6-bp upstream and 6-bp downstream of the codon of interest. Code is available upon request.

For manuscripts utilizing custom algorithms or software that are central to the research but not yet described in published literature, software must be made available to editors and reviewers. We strongly encourage code deposition in a community repository (e.g. GitHub). See the Nature Portfolio guidelines for submitting code & software for further information.

Data

Policy information about availability of data

All manuscripts must include a data availability statement. This statement should provide the following information, where applicable:

- Accession codes, unique identifiers, or web links for publicly available datasets
- A description of any restrictions on data availability
- For clinical datasets or third party data, please ensure that the statement adheres to our policy

The CTX-M-14 Y105W structure information has been deposited in the Protein Data Bank and assigned as PDB ID 7UON. Calculated residue fitness, frequency, k* values, and steady-state kinetics datasets represented in this article are available in Supplementary Data 2. Additionally, sequencing counts following PHRED filtering used to generate fitness and frequency values are available in Supplementary Data 1. Plasmid expression constructs of beta-lactamases are available upon request.

Human research participants

Policy information about studies involving human research participants and Sex and Gender in Research.

Reporting on sex and gender	N/A
Population characteristics	N/A
Recruitment	N/A
Ethics oversight	N/A

Note that full information on the approval of the study protocol must also be provided in the manuscript.

Field-specific reporting

Please select the one below that is the best fit for your research. If you are not sure, read the appropriate sections before making your selection.

☒ Life sciences ☐ Behavioural & social sciences ☐ Ecological, evolutionary & environmental sciences

For a reference copy of the document with all sections, see nature.com/documents/nr-reporting-summary-flat.pdf

Life sciences study design

All studies must disclose on these points even when the disclosure is negative.

Sample size	Sample sizes were not predetermined based on statistical methods. Library selection in the three antibiotic conditions were repeated at least once, with some exceptions. For those exceptions, further validation (via steady-state kinetics and MIC determination) of a subset of mutations within these libraries support our general conclusions. Steady state kinetic determinations are based on many data points including replicates and singly determined values at multiple different substrate concentrations to sufficiently define the Michaelis-Menten curve, and each point is represented in Figure S5. Minimum inhibitory concentrations were also repeated at least twice, as is generally practiced in the field.
Data exclusions	In cases where the number of mutant sequences following beta-lactam antibiotic selection was equal to 0, the replicate was excluded from fitness measurements. In cases where all replicates were excluded, the fitness was not quantifiable and therefore not included, though we can conclude the fitness is very low. Since such a mutant is not represented, we cannot quantify its fitness, but it is understood to be low since it is below the limit of detection based on the number of NGS reads.
Replication	Fitness results were generally repeatable across biological replicates. In cases where a deep sequencing variant produces inconclusive results, no concrete conclusions concerning the variant's fitness are made without further data validation. Kinetics and minimum inhibitory concentration measurements were consistently repeatable.
Randomization	N/A
Blinding	Investigators were not blinded. Blinding during collection was not needed because conditions were well-controlled. Blinding is also not necessary because the results are quantitative and did not require subjective judgment or interpretation. Blinding is not typically used in the field.

Reporting for specific materials, systems and methods

We require information from authors about some types of materials, experimental systems and methods used in many studies. Here, indicate whether each material, system or method listed is relevant to your study. If you are not sure if a list item applies to your research, read the appropriate section before selecting a response.

Materials & experimental systems

n/a	Involved in the study
☒	☐ Antibodies
☒	☐ Eukaryotic cell lines
☒	☐ Palaeontology and archaeology
☒	☐ Animals and other organisms
☒	☐ Clinical data
☒	☐ Dual use research of concern

Methods

n/a	Involved in the study
☒	☐ ChIP-seq
☒	☐ Flow cytometry
☒	☐ MRI-based neuroimaging
